# The structural and functional contributions of β-glucosidase-producing microbial communities to cellulose degradation in composting

**DOI:** 10.1186/s13068-018-1045-8

**Published:** 2018-02-27

**Authors:** Xiangyun Zang, Meiting Liu, Yihong Fan, Jie Xu, Xiuhong Xu, Hongtao Li

**Affiliations:** College of Resources and Environmental Sciences, Northeast Agricultural University, Harbin, 150030 China

**Keywords:** β-Glucosidase, Aerobic composting, Cellulose degradation, Functional microbial community

## Abstract

**Background:**

Compost habitats sustain a vast ensemble of microbes that engender the degradation of cellulose, which is an important part of global carbon cycle. β-Glucosidase is the rate-limiting enzyme of degradation of cellulose. Thus, analysis of regulation of β-glucosidase gene expression in composting is beneficial to a better understanding of cellulose degradation mechanism. Genetic diversity and expression of β-glucosidase-producing microbial communities, and relationships of cellulose degradation, metabolic products and the relative enzyme activity during natural composting and inoculated composting were evaluated.

**Results:**

Compared with natural composting, adding inoculation agent effectively improved the degradation of cellulose, and maintained high level of the carboxymethyl cellulose (CMCase) and β-glucosidase activities in thermophilic phase. Gene expression analysis showed that glycoside hydrolase family 1 (GH1) family of β-glucosidase genes contributed more to β-glucosidase activity in the later thermophilic phase in inoculated compost. In the cooling phase of natural compost, glycoside hydrolase family 3 (GH3) family of β-glucosidase genes contributed more to β-glucosidase activity. Intracellular β-glucosidase activity played a crucial role in the regulation of β-glucosidase gene expression, and upregulation or downregulation was also determined by extracellular concentration of glucose. At sufficiently high glucose concentrations, the functional microbial community in compost was altered, which may contribute to maintaining β-glucosidase activity despite the high glucose content.

**Conclusion:**

This research provides an ecological functional map of microorganisms involved in carbon metabolism in cattle manure–rice straw composting. The performance of the functional microbial groups in the two composting treatments is different, which is related to the cellulase activity and cellulose degradation, respectively.

**Electronic supplementary material:**

The online version of this article (10.1186/s13068-018-1045-8) contains supplementary material, which is available to authorized users.

## Background

Composting is a process involving a complex ecosystem with many interacting factors, in which biodegradable organic wastes are stabilized and converted by the action of some microorganisms under controlled conditions [1, 2]. Microorganisms are the essential factors for the successful operation of composting. To effectively control the composting process, it is necessary to understand the microbial community structure and its change, especially its special role in decomposition of organic matters [3]. In this regard, cellulose the most abundant carbohydrate available from plant biomass, and can be used as a carbon source during composting. All cellulose-degrading organisms possess cellulase systems. Moreover, cellulose degradation is accomplished by the synergistic action among endoglucanases (E.C. 3.2.1.4), cellobiohydrolases (E.C. 3.2.1.91), and β-glucosidases (BGLs, E.C. 3.2.1.21) [4]. Only the first two enzymes act directly, depolymerizing cellulose fiber and releasing final products as oligosaccharides of different sizes, and cellobiose. β-Glucosidase completes the final step of hydrolysis by converting the cellobiose to glucose [5]. β-Glucosidases, which are frequently very sensitive to the presence of glucose, one of the primary products of their catalytic activity [5–7], often play the rate-limiting role in cellulose degradation. This limits their application in commercial scale cellulose degradation ventures [6, 8]. Given the importance of cellulose degradation in providing carbon during the composting process, it is needful to understand the diversity, properties, and expression of the enzymes involved and to identify β-glucosidase enzymes that are insensitive to product inhibition by glucose.

β-Glucosidases are a heterogeneous group of phylogenetically conserved, hydrolytic enzymes widely distributed in the living world. This enzyme family plays a pivotal role in several biological processes [9]. In cellulolytic microorganisms, for example, β-glucosidases are involved in cellulase induction (due to its transglycosylation activities) and cellulose hydrolysis [10]. β-Glucosidases are mostly placed in either family 1 or family 3 of the glycosyl hydrolases, although they are also found in families 5, 9, and 30 of the glycosyl hydrolases [11–13]. Family 1 comprises nearly 62 β-glucosidases from archaebacteria, plants, and mammals, and includes 6-phospho-β-glycosidases and thioglucosidases. Most family 1 enzymes also show significant β-galactosidase activity [14]. Family 3 of the glycosyl hydrolases contains nearly 44 β-glucosidases and hexosaminidases expressed by bacteria, molds, and yeasts. Most of the fungal β-glucosidases belong to family 3 of the glycosyl hydrolases [14].

β-Glucosidases are expressed by a wide range of organisms. For example, β-glucosidase production has been reported in filamentous fungi, such as *Aspergillus niger*, *Aspergillus oryzae*, *Penicillium brasilianum*, *Penicillium decumbens*, *Phanerochaete chrysosporium,* and *Paecilomyces* spp. There are also reports of β-glucosidase production by yeasts (mostly from *Candida* spp.) and a few bacteria [15–17], though microscopic fungi appear to be the most important source [14]. Interestingly, several species of *Aspergilli* are known to produce glucose-tolerant β-glucosidases that are insensitive to product inhibition [18, 19]. β-Glucosidases have attracted considerable attention in recent years due to their important roles in various biotechnological processes such as hydrolysis of isoflavone glucosides, production of fuel ethanol from agricultural residues, release of aromatic compounds from flavorless precursor, etc. [14]. Glucose-tolerant β-glucosidases can circumvent the problem of feedback inhibition in bioethanol production, and it is believed that more glucose-tolerant β-glucosidases may be prevalent in nature, especially in filamentous fungi. Isolating and characterizing these enzymes are likely to help in the design of better enzyme cocktails for biomass hydrolysis, and may help develop targeted approaches for modifying the glucose tolerance of existing β-glucosidases [14].

Previous studies have investigated β-glucosidase in environmental samples and mostly have focused on cultured microbes [20]. However, less than 1% of microorganisms are culturable under laboratory conditions. Recently, cellulolytic diversity of β-glucosidases and their functions were analyzed in composite environmental samples, such as those gathered during the composting process [21, 22], during the traditional Chinese cereal starter solid-state fermentation process [23] and from soil samples [24]. However, these researches were still not sufficient for a clear understanding of the relationship between β-glucosidase-producing microbial communities and the degradation of cellulose in composite environmental samples.

In this study, we investigated the genetic diversity and expression of β-glucosidase-producing microbial communities, and relationships between cellulose degradation, metabolic products, and the relative enzyme activity in different phases of composting in the presence and absence of inoculants. This research provides an ecological functional map of microorganisms involved in carbon metabolism in cattle manure-rice straw composting.

## Methods

### Composting process and compost samples

The aerobic composting of cattle manure-rice straw was performed at the College of Resources and Environmental Sciences of Northeast Agricultural University in China. First, cattle manure was moistened with water to achieve a 60% moisture content. Rice straw was moistened with 1% lime water to soften the straw tissue and improve its water retention capacity. Rice straw, cattle mature, urea, calcium superphosphate, and gypsum at a ratio of 90:60:1.5:2.5:3 (wt/wt/wt/wt), respectively, were mixed with an initial C/N ratio of approximately 32:1. The details of the physico-chemical properties of straw-cattle manure compost are described in Xu [25].

Two pyramidal stacks (2 m in diameter and 1.5 m in height) were built with the mixed materials mentioned above for composting. Lignocellulose-decomposing inoculants (named DN-1) were added to one stack (inoculated compost), and the other control stack did not receive any inoculants (natural compost). The lignocellulolytic inoculants (DN-1) included *Phanerochaete chrysosporium*, *Streptomyces griseorubens* C-5, *Bacillus subtilis* W1, *Bacillus methylotrophicus* W8, and *Bacillus amyloliquefaciens* X9. *P. chrysosporium* was purchased from China General Microbiological Culture Collection Center (CGMCC). *S. griseorubens* C-5 was isolated from soil in Heilongjiang Province of China. *Bacillus subtilis* W1; *Bacillus methylotrophicus* W8 and *Bacillus amyloliquefaciens* X9 were isolated from fresh cattle manure [26, 27]. Cellulase, laccase, peroxidase, xylanase, amylase, and protease activities were observed in the microorganisms of the inoculants [26, 27]. For the inoculation, the suspension of the inoculants [1 × 10^9^ colony-forming units (CFU) mL^−1^] were evenly sprayed onto composting substrate at the rate of 10 mL of the suspension versus 1 kg compost. The composting process lasted approximately 46 days, and the piles were turned manually twice a week during the first 2 weeks and then once a week afterward to maintain aeration. Samples were collected from three locations within the stacks (15, 50, and 90 from the top surface of the pile), mixed, and then stored at − 80 °C on days 1, 3, 12, 18, 22, 31, and 46.

### Enzymatic activities, degradation of lignocellulose, content of glucose, and cellobiose analysis

Ten grams of sample were transferred to a flask containing 50 mL acetate buffer (0.1 M, pH 5.0). The flask was shaken at 200 r min^−1^ for 1 h. The homogenate was centrifuged (1315*g*) at 4 °C for 20 min, and the supernatant was filtered through filter papers (Whatman No. 1) and then used for measurement of enzymatic activity. The assay of CMCase activity was carried out by measuring the reducing sugars by the method as described by Zeng [28]. The activity of β-glucosidase was measured using *p*-nitrophenyl β-d-glucoside (PNPG) as described by Herr [29]. The intracellular β-glucosidase fraction was estimated in the supernatant fraction of a portion of the acid-treated compost samples with glass beads, and then using *p*-nitrophenyl β-d-glucoside (PNPG) as described by Herr [29].

The glucose and cellobiose contents in the samples were determined with high-performance liquid chromatography (HPLC), using the Bio-Rad Aminex HPX-87H chromatographic column (Bio-Rad, America) with 0.005 M H_2_SO_4_ as the mobile phase, a column temperature of 60 °C, and a velocity of 0.5 mL min^−1^, as assessed by a refractive index detector.

The cellulose, hemicellulose, and lignin contents were determined with an ANKOM220 fiber analyzer using the method described by Van [30]. Briefly, the hemicellulose content was estimated as the difference between the neutral-detergent fiber (NDF) and the acid-detergent fiber (ADF); the cellulose content was estimated as the difference between the ADF and the acid-detergent lignin (ADL); and the lignin content was estimated as the difference between the ADL and the ash content.

### DNA and cDNA preparation

DNA was extracted from compost samples (3 g) using a procedure described previously by Liu et al. [31]. After DNA extraction, the crude DNA was purified using the E.Z.N.A. Gel Extraction Kit (Omega Bio-Tek, Inc., Georgia, USA). Total RNA was extracted from the samples using an RNA PowerSoil total RNA isolation kit (MoBio, Inc., Carlsbad, CA USA) according to the manufacturer’s guidelines.

cDNA was generated from the total RNA using a PrimeScript™ RT Reagent Kit with gDNA Eraser (Perfect Real Time) (Takara Bio, Inc., Japan) according to the manufacturer’s guidelines. Random hexamers were used as primers during the first strand synthesis. RT Primer Mix, which contains an Oligo dT Primer and Random hexamers, was used during the second strand synthesis.

### PCR-DGGE analysis of the GH1B and GH3E expression

Standard PCR reaction mixtures were used for all primer sets. The primers and thermal cycling conditions used are shown in Table 1 and for more information on primer design and detection of β-glucosidase genes, see Li [22]. The PCR products were analyzed on a 1% agarose gel electrophoresis before DGGE analysis. DGGE was performed with the D-gene system (Bio-Rad Laboratories, Hercules, CA, USA). Bands chosen for sequencing were excised from the DGGE gel, placed into PCR tubes, resuspended in 30 μL sterile H_2_O, and maintained at 4 °C overnight. These tubes were then used for reamplification of the DNA in the bands with primer pair. After reamplification, PCR products were rerun on DGGE to verify their purity and to test for heteroduplexes. The suitable PCR products were sent to Beijing Huada Gene Company (Beijing, PR China) for sequencing.

**Table 1:** Primers used for PCR-DGGE and thermal denaturation conditions of the primers

Primer	Sequences (5′–3′)	Thermal conditions
GH1 family gene (bacteria)		
GH1BF	CCT ACC AGA TYC ARG G	
GH1BR	GAG GAA GRT CCC ART G	95 °C, 30 s, one cycle, 95 °C for 5 s, 50 °C for 30 s, 72 °C for 60 s, 45 cycles
GH1BF-GC	CCT ACC AGA TYG ARG GCGC CCG CCG CGC GCG GCG GGC GGG GCG GGG GCA CGG G	
GH3 family gene (fungi)		
GH3EF	GGT GGT CGC RRY TGG GA	
GH3ER	CCA GGC ATC GGW CAT RTC	95 °C, 30 s, one cycle, 95 °C for 5 s, 56 °C for 30 s, 72 °C for 60 s, 45 cycles
GH3ER-GC	CGC CCG CCG CGC GCG GCG GGC GGG GCG GGG GCA CGG GGG TGG TCG CRR YTG GGA	

Modified bases: Y = CT, R = AG, W = TA

### Real-time PCR quantification (qPCR) of β-glucosidase genes

From each of the DGGE β-glucosidase gene profiles, six bands were selected according to the nucleotide sequences with the highest similarity in the database. The abundance of bacterial GH1 family β-glucosidase genes and fungal GH3 family β-glucosidase genes, as well as the abundance of individual bacterial (GH1B-a1, GH1B-b1, GH1B-c1, and GH1B-d1) and fungal (GH3E-d3 and GH3E-e3) taxa during the composting process were measured by quantification of the respective β-glucosidase genes. For this purpose, gene-specific primers were designed using the Primer 5.0 software and the Primer-BLAST in NCBI (National Center for Biotechnology Information). The GenBank accession numbers for the nucleotide sequences of some of the species isolated in this study are listed in Table 2 and the gene-specific primers sequences are shown in Table 3.

**Table 2:** GenBank accession numbers and similarities of the most homologous sequences to the nucleotide sequences isolated from the DGGE bands selected in this study

Clone no.	Most similar known sequence in GenBank	GenBank no.	Similarity (%)
GH1B-a1	*Bacillus* sp.	AB009410.1	70
GH1B-b1	*Devosia* sp.	KP663811.1	73
GH1B-c1	*Streptomyces* sp.	JX032780.1	73
GH1B-d1	*Microbacterium*	JX960412.1	83
GH3E-d3	*Aspergillus niger*	Q9P8F4	83
GH3E-e3	*Penicillium brasilianum*	EF527403	97

**Table 3:** Universal and specific primers of β-glucosidase genes for real-time quantitative PCR

Primer	Sequences (5′–3′)	Primer	Sequences (5′–3′)
GH1 family genes (bacteria)		*Microbacterium*	
GH1BF	CCT ACC AGA TYC ARG G	GH1B-d1F	ACGACCATTGACCAGACGGGAGT
GH1BR	GAG GAA GRT CCC ART G	GH1B-d1R	GAAGGAGCGGCTGACGAGGAC
*Bacillus* sp.		GH3 family genes (fungi)	
GH1B-a1F	AGATTGAAGGAGCAAGGAATGAGG	GH3EF	GGT GGT CGC RRY TGG GA
GH1B-a1R	AGGAAGGTCCCAGTGGTAGAGC	GH3ER	CCA GGC ATC GGW CAT RTC
*Devosia* sp.		*Aspergillus niger*	
GH1B-b1F	ACCAGAACGAAGGCGGGCAGAC	GH3E-d3F	GGCAAGGCTATGGGTCAG
GH1B-b1R	CCAGTCATAGAGCGTGGAGTAG	GH3E-d3R	CCAGCGGAAATGCTCTAAT
*Streptomyces* sp.		*Penicillium*	
GH1B-c1F	CGAGCATACACTGGGCAACA	GH3E-e3F	TATGGGTGGTGATGTGAAT
GH1B-c1R	GAAGGTTVVAATGGCACAGCGT	GH3E-e3R	CAAGTGGCTGGTTGAGTT

DNA and RNA served as templates for the quantitative analysis of gene abundance and expression, respectively. qPCR was conducted using SYBR^®^ Premix Ex Taq™ II (Perfect Real Time) (Takara Bio, Inc., Japan) in a typical 20 μL PCR mixture; the reaction conditions are listed in Tables 1 and 3. Then, the samples were run on the Applied Biosystems 7500/7500 fast real-time PCR systems (Applied Biosystems, USA) [26]. qPCR of β-glucosidase genes was performed in triplicate as described in the Additional file 4. Melting curve analysis and agarose gel electrophoresis confirmed the specificity of the amplification.

### Statistical analysis

SPSS 13.0 for windows was used for the statistical analysis. One-way repeated measures ANOVA was used to test differences in the measured parameters during composting, and post hoc Tukey’s test was used to further investigate these differences (*p* < 0.05). Graphpad Prism 6 (GraphPad Software, Inc., USA) was used for the data analysis and draw data charts.

## Results

### Temperature evolution during composting

**Fig. 1:**
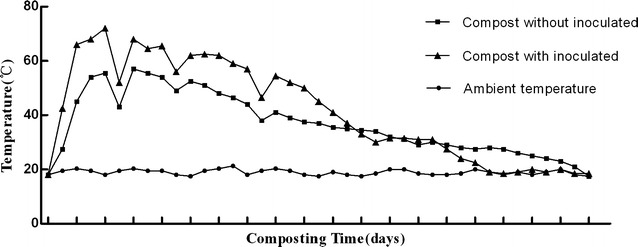
Changes in temperature of the two aerobic composting piles

The composting process was divided into three phases base on temperature, including initial phase (days 1–3), thermophilic phase (days 3–18 for natural compost, days 3–22 for inoculated compost) and cooling phase (days 18–46 for natural compost, days 22–46 for inoculated compost) (Fig. 1). In natural compost, the temperature reached thermophilic phase (> 45 °C) on the fifth day and decreased to the ambient temperature after 46 days of composting. For inoculated compost, the general profile of temperature during composting was similar to that of the natural compost, but the whole temperature level was obviously much higher. Temperature increased rapidly during the initial stage of composting, going into the thermophilic phase (> 45 °C) on the third day, and reaching a maximal value of 72 °C on the fifth day. Thereafter, temperature decreased gradually, and after 35 days of composting, it decreased to the ambient temperature (Fig. 1). The dynamic of temperature in inoculated compost was consistent with the observations of Jurado’s [32, 33], and this can be explained by the fact that the introduction of abundant exogenous microorganisms with various decomposing enzymes may accelerate the microbial metabolism, resulting in a higher temperature in the compost and finally shortening the compost maturity process [34, 35].

### Degradation of lignocellulose during composting

**Fig. 2:**
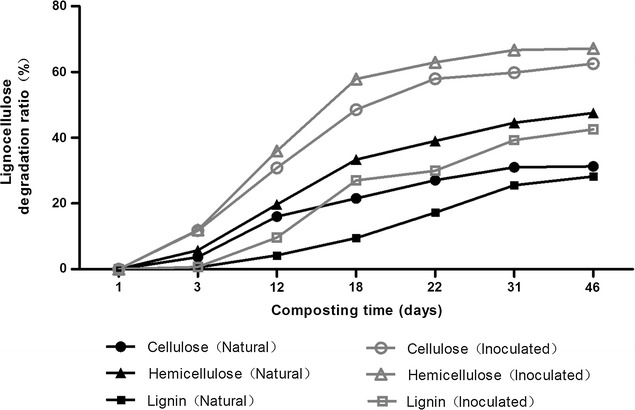
Degradation ratio of cellulose, hemicellulose and lignin during the two aerobic compost piles

During the initial stage of composting in the natural compost, cellulose, hemicellulose, and lignin degraded slowly, with degradation rates of only 3.69, 5.78, and 0.58% after 3 days of composting, respectively (Fig. 2). In contrast, the degradation ratios of cellulose, hemicellulose, and lignin in the inoculated compost reached 11.78, 11.97, and 0.73%, respectively, during the same period. By the end of the composting process, degradation rates of cellulose, hemicellulose, and lignin were significantly higher (*p* < 0.05) in inoculated compost, reaching 62.57, 67.14, and 42.54%, respectively, on day 46. This suggests that inoculants were effective to accelerate the degradation of lignocellulose during composting and, therefore, enhanced the whole composting process eventually, which was consistent with the results by Jurado and Tuomela [32, 33, 36].

### Dynamic of cellobiose and glucose during composting

**Fig. 3:**
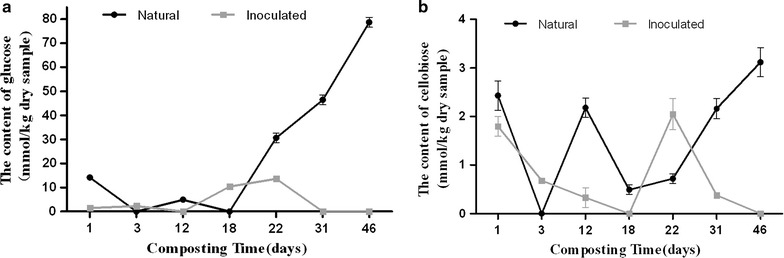
Change in content of glucose during natural and inoculated composts (**a**); the content of cellobiose during natural and inoculated composts (**b**)

In the natural compost, during the first 3 days, the dynamic of contents of glucose and cellobiose showed similar trends (Fig. 3) which declined sharply, and none of either substance was detected on day 3. From then on, contents of glucose and cellobiose increased, to the first peak (4.90 and 2.18 mmol kg^−1^, respectively) on day 12, and then declined until day 18. After day 18, glucose and cellobiose accumulated gradually in compost until the end of composting with the maximum values of 78.70 and 3.10 mmol kg^−1^ on day 46, respectively. Content of glucose kept at a low level in the inoculated compost during the first 12 days and reached a higher level from day 18 to day 22. Content of cellobiose in the inoculated compost declined continuously before day 18 with no cellobiose detected on day 18. From day 18 forward, the content increased and reached maximum (2.05 mmol kg^−1^) on day 22 (Fig. 3).

### Dynamic of β-glucosidase activity and CMCase activity during composting

**Fig. 4:**
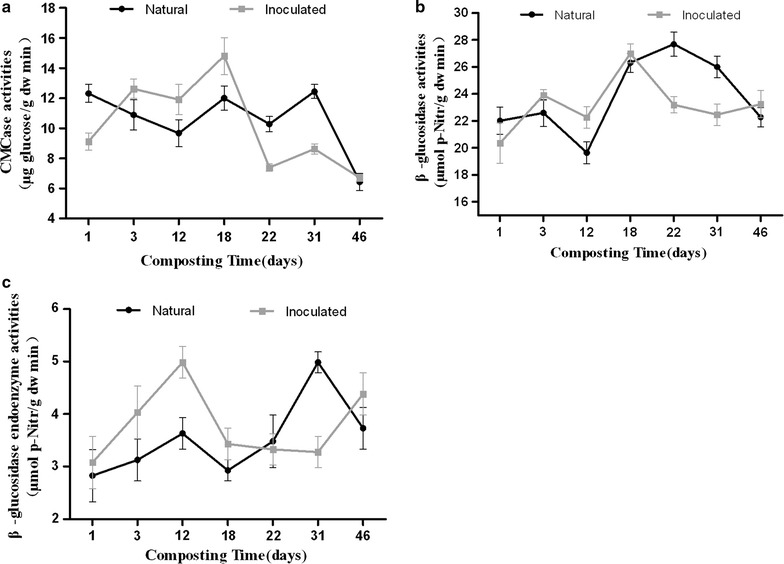
Dynamics of CMCase activity (**a**), β-glucosidase activity (**b**), and β-glucosidase endoenzyme activity (**c**) during natural and inoculated composts

In natural compost, CMCase activity remained steady from day 1 to day 31 of composting (Fig. 4a), and then declined until the end of composting. In general, β-glucosidase activity increased from day 1 to day 22 except for a drop on day 12; it thereafter declined (Fig. 4b). Intracellular β-glucosidase activity kept rising from day 1 to day 31 with two peaks appearing on day 12 and day 31, and then dropped (Fig. 4c). In inoculated compost, both CMCase activity and β-glucosidase activity increased during the first 18 days of composting and then declined until the end. For intracellular β-glucosidase, the maximum activity was observed on day 12. CMCase activity, β-glucosidase activity, and intracellular β-glucosidase activity in inoculated compost were higher than those in natural compost during thermophilic phase of composting. In the contrary, lower CMCase activity, β-glucosidase activity, and intracellular β-glucosidase activity were observed during cooling phase in inoculated compost.

### PCR-DGGE analysis of the composition of the functional microbial community during composting

**Fig. 5:**
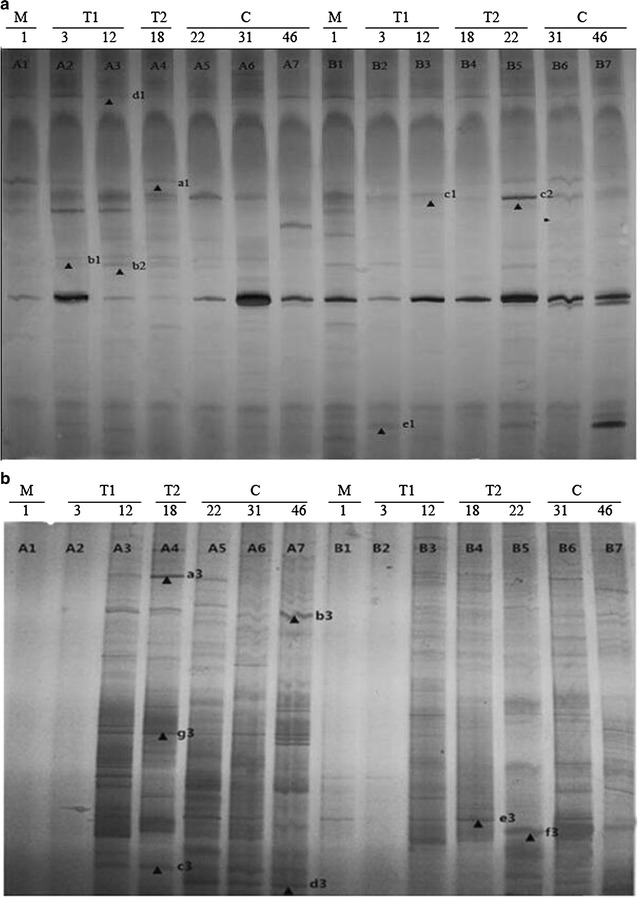
Diversity of bacterial GH1 family and fungal GH3 family β-glucosidase genes in the microbial communities present during natural (lanes A1–7) and inoculated (lanes B1–7) composting as shown by DGGE of PCR products amplified using the GH1F/GH1R-GC primer pair (**a**) or the GH3E/GH3ER-GC primer pair (**b**). a1–g1 and a3–g3 correspond to fourteen unique bands identified as the dominant species during the composting process. Lane numbers correspond to the sampling days. Letters indicate the different stages of the composting process: the mesophilic phase (M), the initial stage of thermophilic phase (T1), the later stage of the thermophilic phase (T2), and the cooling phase (C)

The DGGE profiles of the GH1 β-glucosidase genes from bacteria showed that higher abundance and diversity of β-glucosidase genes were found in the natural compost than in the inoculated compost during the thermophilic phase (Fig. 5a). However, higher abundance and diversity of bacterial community were observed in the inoculated compost during the cooling phase, and there was an obvious change in the bacterial community structure during this phase in both composts. A few GH3 β-glucosidase genes from fungi were detected at the initial phase of composting of both composts (Fig. 5b). During thermophilic phase, abundance and diversity of those fungal genes increased. The presence of abundant β-glucosidase gene bands indicated that fungal community diversity and β-glucosidase genes abundance were higher in cooling phase than other phases in both composts. The fungal GH3 family β-glucosidase gene DGGE profiles indicated that β-glucosidase genes abundance and fungal diversity in the natural compost were higher than that in inoculated compost (Fig. 5b).

### β-Glucosidase gene characterization and quantification

**Fig. 6:**
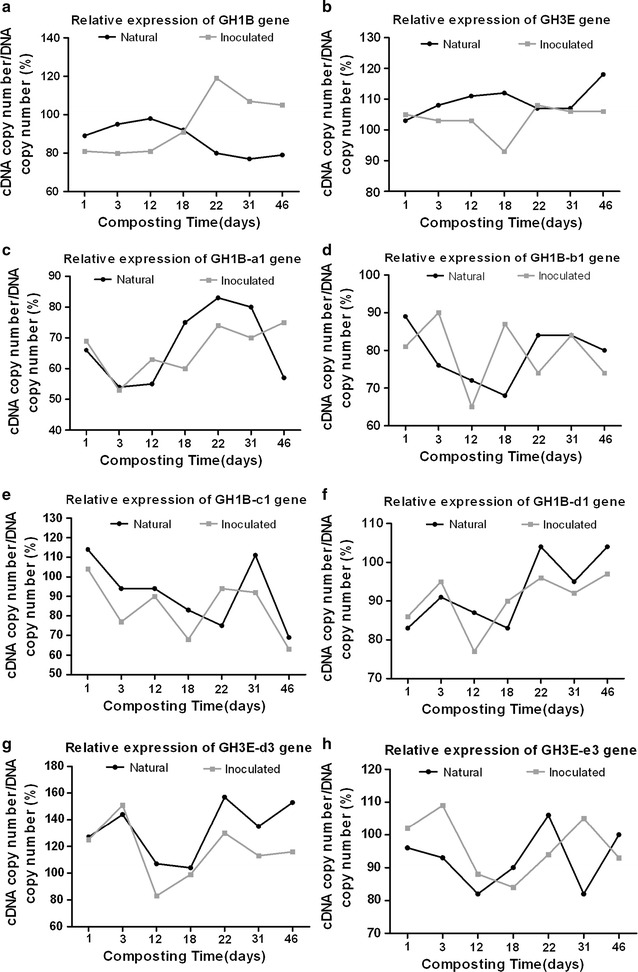
Differences in the relative expression of family 1 β-glucosidase genes from bacteria (GH1) (GH1B (**a**), GH1B-a1 (**c**), GH1B-b1 (**d**), GH1B-c1 (**e**) and GH1B-d1 (**f**) genes) and family 3 β-glucosidase genes from fungi (GH3) (GH3E (**b**), GH3E-d3 (**g**) and GH3E-e3 (**h**) genes) in the natural composting and the inoculated composting

Abundance of bacterial and fungal β-glucosidase-encoding genes was measured by quantifying the β-glucosidase gene copy number as well as the expression level during the aerobic composting. Based on the DNA level, different patterns of abundance of bacterial β-glucosidase-encoding genes (GH1B) were found in natural compost and inoculated compost during composting (Additional file 1: Figure S1a). Gene copy number was higher in natural compost during thermophilic phase than in inoculated compost; however, lower number of gene copy was detected during cooling phase in the samples from natural compost (Additional file 1: Figure S1a). The dynamic of copy number of cDNA of GH1B gene was different from that of gene copy number (Additional file 1: Figure S1b). Copy number of cDNA of GH1B gene increased slightly during both natural and inoculated composting, respectively, reaching 8.15 and 8.17 log copies g^−1^ on day 46; the lowest values appeared on day 18 for natural compost (7.41 log copies g^−1^) and on day 12 for inoculated compost (7.26 log copies g^−1^). The ratio of cDNA/DNA of GH1B gene was higher in the natural compost in thermophilic phase than in the cooling phase, whilst for the inoculated compost, converse facts were observed (Fig. 6a).

Fungal β-glucosidase-encoding gene (GH3E) abundance remained at a relatively stable level during the whole composting process both in natural (8.03 log copies g^−1^) and inoculated (9.48 log copies g^−1^) on day 18 (Additional file 1: Figure S1c). In general, copy number of cDNA of GH3E gene increased in both natural and inoculated compost during the entire composting process (Additional file 1: Figure S1d). Interestingly, on day 12, a higher value of cDNA copy number was observed in samples of natural compost, but a lower value was observed in inoculated compost. Similar phenomenon was also found in the dynamic of bacterial cDNA copy number (Additional file 1: Figure S1b). It is noteworthy that the cDNA copy number and transcription efficiency of GH3E gene were higher in natural compost than in inoculated compost in thermophilic phase of composting (Fig. 6b and Additional file 1: Figure S1d).

Despite some fluctuations, generally, cDNA copy number and transcription efficiency of GH1B-a1, GH1B-b1, GH1B-c1, and GH1B-d1 genes showed similar patterns in thermophilic phase (Fig. 6 and Additional file 2: Figure S2), which were consistent with those of GH1B genes (Additional file 1: Figure S1b) both in natural and in inoculated composts. However, in cooling phase, the cDNA copy number and transcription efficiency were different from those of GH1B gene. cDNA copy number and transcription efficiency of GH3E-d3 and GH3E-e3 genes showed similar trends with GH3E gene in the both composts during composting process and exhibited a higher level in cooling phase than in thermophilic phase (Fig. 6 and Additional file 3: Figure S3).

## Discussion

For decades, linking microbial community composition and function in the natural environment had been challenging. Nowadays, however, the availability of an increasing amount of quantitative analyses of the expression of functional gene and the relevant physical as well as chemical properties in environmental samples provide one way to link microbial community composition and function traits. It is well known that the β-glucosidase is the rate-limiting enzyme of degradation of cellulose. Therefore, analysis of regulation of β-glucosidase gene expression in composting could characterize the degradation of cellulose. Metagenomic analysis of microbial consortia enriched from compost showed that β-glucosidases are mostly present in GH3 and GH1 [37]. In this study, expression of GH1 and GH3 family β-glucosidase-encoding genes related to the degradation of cellulose was investigated in natural and inoculated composting. The results showed that inoculating agents caused differences between the two composts, including degradation efficiencies of cellulose and functional microbial activity across the mesophilic phase, the thermophilic phase, and the cooling phase. These report advances existing knowledge by showing that microbial communities have the metabolic potential to utilize cellulose and the byproducts of cellulose degradation in compost environment.

The results of this study further confirmed that cellulose degradation largely occurred in the thermophilic phase of inoculated compost (Fig. 2). In addition, the corresponding CMCase and β-glucosidase activities were relatively higher in the thermophilic phase of inoculated compost, which could explain the higher efficiency of cellulose degradation than natural compost.

Interestingly, from days 3 to 18, cellobiose content was significantly higher in natural compost than in inoculated compost in the thermophilic phase (Fig. 4). Cellobiose was generally considered as an inducer to induction of cellulase genes. Cellobiose and its transglycosylation products: sophorose and gentiobiose were considered the “true” inducers due to their strong inductive effects on cellulase expression in *P. decumbens*, *T. reesei*, and *Penicillium purpurogenum* [15–17, 38]. In this study, the transcription efficiency of the bacterial and fungal β-glucosidase genes was higher in nature compost than in inoculant compost samples (Fig. 6a, b). This could be explained by the fact that cellobiose accumulation achieved a peak in natural compost on day 12 of composting, which might induce the transcription of β-glucosidases genes. In addition, the transcription levels of the bacterial and fungal β-glucosidase genes in natural compost were higher than those in inoculated compost (Additional file 1: Figure S1). Meanwhile, higher levels of transcription of β-glucosidases genes were also found in other individual community members, such as for GH1B-c1, GH1B-d1, and GH3E-d3 in natural compost than in inoculated compost (Additional files 2: Figure S2, Additional file 3: Figure S3). Although up-regulation of expression of β-glucosidase genes occurred in natural compost on day 12, the up-regulated level of enzyme activity was still lower than that in inoculated compost (Fig. 4b, c). We speculated that GH1B-a1 (Additional file 2: Figure S2b), which showed higher transcription effectiveness in inoculated compost, and the other β-glucosidase genes, which were not scanned in this experiment, might contribute more in enzyme activity of β-glucosidase. In addition, the higher temperature in inoculated compost could also play an important role in improving enzyme activity of β-glucosidase. Hollister et al. [39] found that the thermophilic metagenome was significantly enriched in genes related to the uptake of cellobiose compared with mesophilic metagenome, indicating that high temperature could facilitate the uptake of cellobiose. In addition, intracellular cellobiose was more effective in inducing cellulase than extracellular hydrolysis [40]. In this study, it was observed that intracellular β-glucosidase activity in inoculated compost was higher than that in natural compost, suggesting that higher potential transglycosylation activity existed in inoculated compost (Fig. 4c).

In natural compost, most of β-glucosidase genes performed with high transcription level and transcription efficiency, but with low abundance. This phenomenon was found in Pathan’s report that some β-glucosidase genes with low abundance in DNA showed high transcriptional activity, which indicated the importance of these low abundance taxa in cellobiose utilization [41]. However, in inoculated compost, the functional genes performed with high abundance but low transcription efficiency. Based on the observation above, it is concluded that the microbial community presented in inoculated compost could produce cellulase and utilize cellobiose more efficiently.

In this experiment, bacterial GH1 family β-glucosidase genes contributed more to β-glucosidase activity than fungal GH3 family β-glucosidase genes in the later thermophilic phase (from days 18 to 22) in the inoculated compost. Furthermore, at the later stages of the thermophilic phase, in nature compost and inoculant compost, the temperature was over 45 and 60 °C, respectively (Fig. 1). By this time, the transcription efficiency and gene abundance were at the lowest level for both the overall fungal β-glucosidase gene pool, as well as for that of individual community members (GH3E-d3 and GH3E-e3) in both composts (Fig. 6 and Additional file 1: Figure S1, Additional file 3: Figure S3). Conversely, the transcriptional efficiency of GH1B-a1 and GH1B-c1 genes was significantly elevated in the thermophilic phase of inoculated compost, which might be due to the thermophilic nature of these bacteria [42, 43]. A similar phenomenon was found by Simmons [44], where it was shown that several GH family 1 proteins (that is, bacterial genes) were significantly overexpressed in the thermophilic community, whereas GH family 3 (predominantly fungal) genes were significantly overexpressed in the mesophilic community.

In the cooling phase of the natural compost (day 18–day 46), the CMCase and β-glucosidase activities maintained at stable high level (Fig. 4a, b). Cellobiose and glucose were produced and accumulated, and concentration of both reached the maximum on day 46 (Fig. 3). In inoculated compost, the accumulation of cellobiose and glucose occurred from days 18 to 22, but on day 46, cellobiose and glucose were not detected in microbial metabolism (Fig. 3). Based on the observations above, it is reasonable to infer that the degradation of cellulose was conducted smoothly in inoculated compost. On the contrary, this process was disturbed in natural compost.

The β-glucosidase genes from GH family 3, which showed higher transcription levels (Additional file 1: Figure S1d), may be functionally important in the cooling phase of the natural compost. In natural compost, expression of GH3E-d3 and GH3E-e3 were generally up-regulated from days 18 to 46, except for temporary down-regulation from days 22 to 31 (Fig. 6g, h). The down-regulation could be explained by high level of intracellular β-glucosidase activity occurring in the same period (days 22–31) (Fig. 4c), indicating that intracellular β-glucosidase activity played a very crucial role in the regulation of β-glucosidase gene expression. In the report of Berry et al. [45], action of intracellular β-glucosidase IG-1 of *Monilia* sp, which caused the accumulation of glucose within the cell, was supposed to lead to the repression of cellulase production. However, high level of intracellular β-glucosidase activity was associated with up-regulation of transcriptional activity on day 12 in natural compost (Fig. 6a). This up-regulation was possibly caused by the low concentration of extracellular glucose. Therefore, it is proposed that the regulation of expression of β-glucosidase genes was determined not only by intracellular β-glucosidase activity but also by concentration of extracellular glucose.

β-Glucosidase activity can be affected by many factors during the degradation of cellulose to glucose. Several studies have shown that most β-glucosidases are sensitive to glucose, and can be inhibited by the presence of 0.5 and 100 mM glucose due to product feedback [4, 46–48]. Our results showed that high concentrations of glucose in natural compost were associated with a decrease in β-glucosidase activity (Fig. 4b), which is in accordance with the findings of previous researchers.

Based on the observation that Trp168 and Leu173 were conserved in glucose-tolerant GH1 enzymes using Giuseppe’s method [49], we found that the proportion of glucose-tolerant GH1B genes encoding glucose-tolerant β-glucosidases increased from 56 to 77.78% (unpublished data) from day 22 to day 46 in natural compost with the increase of concentration of glucose. The translational efficiency of the GHIB-d1 gene showed a tendency of increase in natural compost from day 31 to day 46 (Fig. 6). β-Glucosidases from *Derosia* spp. were reported to be glucose-tolerant [21]. However, the translational efficiency of non-sugar tolerant genes was significantly inhibited, such as GH1B-a1 and GHIB-c1 (Fig. 6). This finding was consistent with the work of Pérezpons who reported that β-glucosidase activity of *Streptomyces* sp. QM-B814 was inhibited in the presence of glucose [50]. The results suggested that, at sufficiently high glucose concentrations, the functional microbial community was altered and contributes to maintaining the operation of the enzyme despite the high glucose content.

## Conclusions

In this study, the microbial community presented in inoculated compost could produce cellulase and degrade cellulose more efficiently. Intracellular β-glucosidase activity played a crucial role in regulation of β-glucosidase gene expression, and the up-regulation or down-regulation was also determined by concentration of extracellular glucose. At sufficiently high glucose concentrations, the functional microbial community in compost was altered, which may contribute to maintaining β-glucosidase activity despite the high glucose content. These results provide an efficient approach to more efficiently degrade cellulose in compost, thereby enhancing the activity of β-glucosidase by promoting β-glucosidase-encoding genes expression.

## Additional files

**Additional file 1: Figure S1.** Differences in the abundance and expression of GH1B and GH3E genes in the natural compost and the inoculated compost.

**Additional file 2: Figure S2.** Differences in the abundance and expression of family 1 β-glucosidase genes from bacteria (GH1) in the natural compost and the inoculated compost.

**Additional file 3: Figure S3.** Differences in the abundance and expression of family 3 β-glucosidase genes from fungi (GH3) in the natural compost and the inoculated compost.

**Additional file 4.** Additional Materials and Methods.
