# Supplementary material for: The structural and functional contributions of β-glucosidase-producing microbial communities to cellulose degradation in composting
Source: Biotechnol Biofuels. 2018 Feb 27;11:51. doi: 10.1186/s13068-018-1045-8 (PMC5828080; doi:10.1186/s13068-018-1045-8)
Supplement: Supplementary file 1 — Additional file 1: Figure S1. Differences in the abundance and expression of GH1B and GH3E genes in the natural compost and the inoculated compost. [file 13068_2018_1045_MOESM1_ESM.docx]

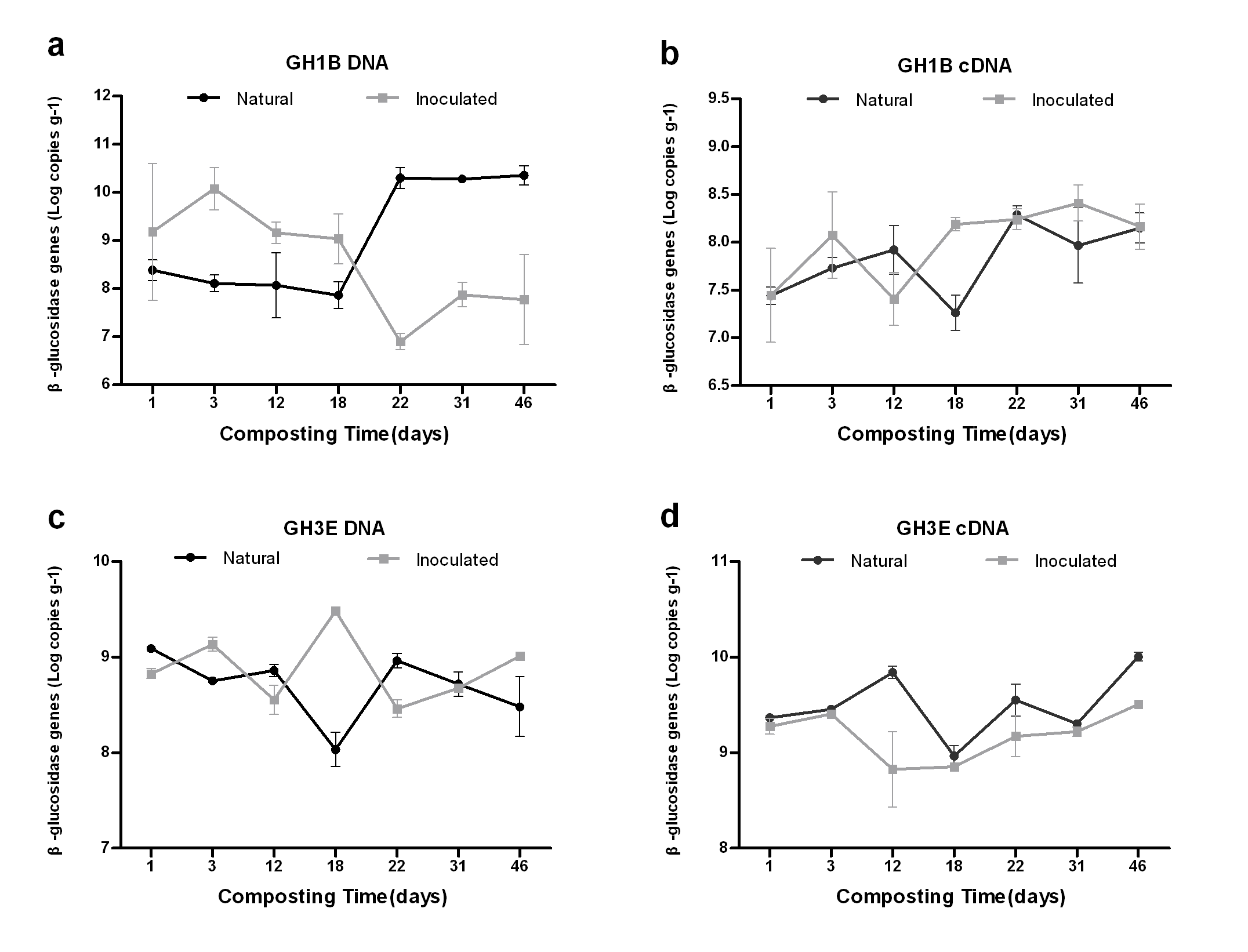


Additional file 1: Figure. S1. Differences in the abundance and expression of GH1B and GH3E genes in the natural compost and the inoculated compost.
