# Supplementary material for: The structural and functional contributions of β-glucosidase-producing microbial communities to cellulose degradation in composting
Source: Biotechnol Biofuels. 2018 Feb 27;11:51. doi: 10.1186/s13068-018-1045-8 (PMC5828080; doi:10.1186/s13068-018-1045-8)
Supplement: Supplementary file 2 — Additional file 2: Figure S2. Differences in the abundance and expression of family 1 β-glucosidase genes from bacteria (GH1) in the natural compost and the inoculated compost. [file 13068_2018_1045_MOESM2_ESM.docx]

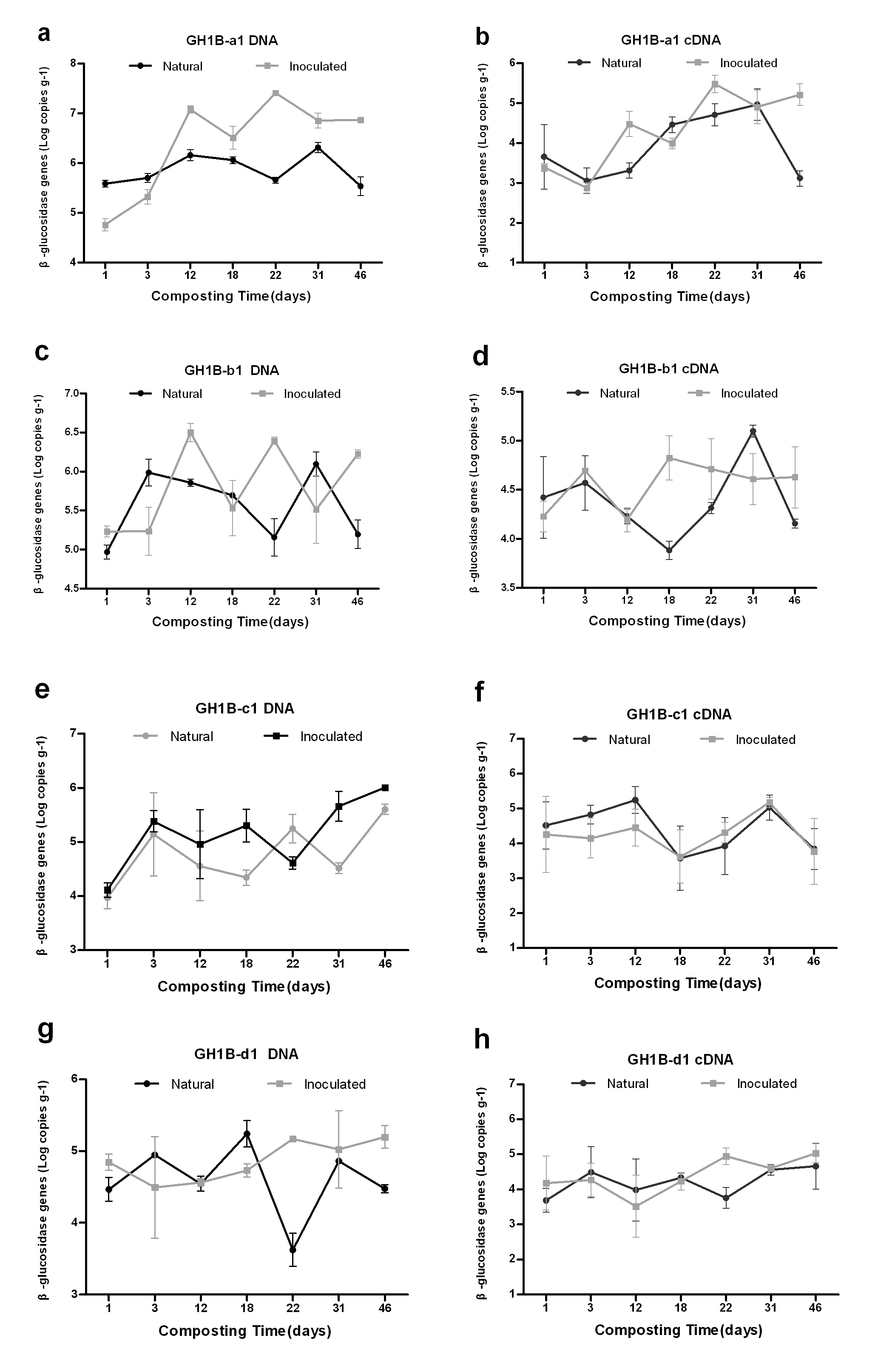


Additional file 2: Figure. S2. Differences in the abundance and expression of family 1 β-glucosidase genes from bacteria (GH1) in the natural compost and the inoculated compost.
