# Supplementary material for: The structural and functional contributions of β-glucosidase-producing microbial communities to cellulose degradation in composting
Source: Biotechnol Biofuels. 2018 Feb 27;11:51. doi: 10.1186/s13068-018-1045-8 (PMC5828080; doi:10.1186/s13068-018-1045-8)
Supplement: Supplementary file 3 — Additional file 3: Figure S3. Differences in the abundance and expression of family 3 β-glucosidase genes from fungi (GH3) in the natural compost and the inoculated compost. [file 13068_2018_1045_MOESM3_ESM.docx]

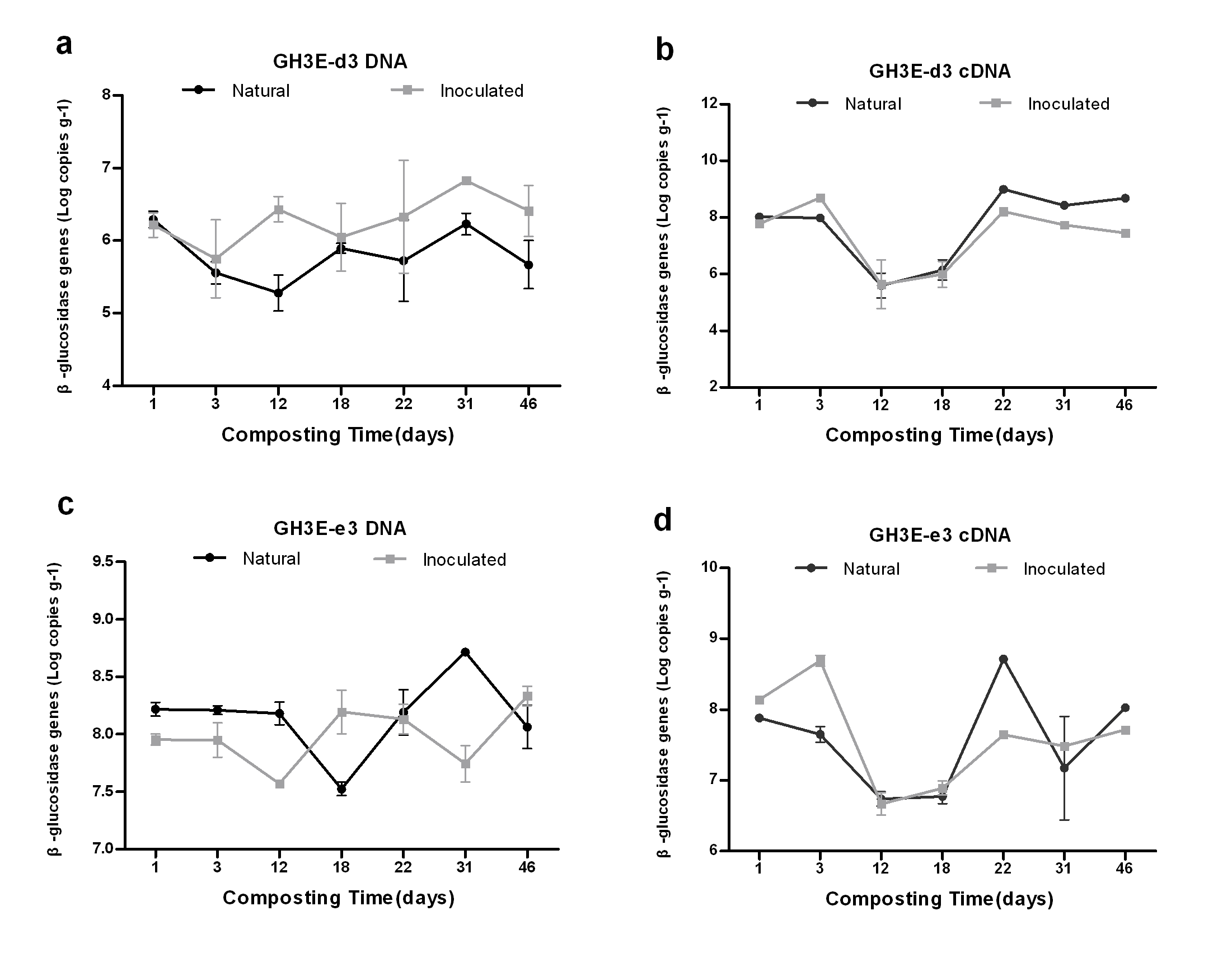


Additional file 3: Figure. S3. Differences in the abundance and expression of family 3 β-glucosidase genes from fungi (GH3) in the natural compost and the inoculated compost.
