# Supplementary material for: The structural and functional contributions of β-glucosidase-producing microbial communities to cellulose degradation in composting
Source: Biotechnol Biofuels. 2018 Feb 27;11:51. doi: 10.1186/s13068-018-1045-8 (PMC5828080; doi:10.1186/s13068-018-1045-8)
Supplement: Supplementary file 4 — Additional file 4. Additional Materials and Methods. [file 13068_2018_1045_MOESM4_ESM.docx]

**The structural and functional contributions of β-glucosidase-producing microbial communities to cellulose degradation in composting**

Xiangyun Zang , Meiting Liu, Yihong Fan, Jie Xu, Xiuhong Xu, Hongtao Li

College of Resources and Environmental Sciences, Northeast Agricultural University, Harbin 150030, China. Correspondence and requests for materials should be addressed to X.X. (email: xuxiuhong@neau.edu.cn) or to H.L. (email: hongtaoli@neau.edu.cn).

Additional Materials and Methods

**Real-Time PCR quantification (qPCR) of β-glucosidase genes**

qPCR of β-glucosidase genes was performed in triplicate using the SYBR^®^ Premix Ex Taq^TM^ II (Takara Bio, Inc., Japan) and an ABI 7500 Fast Real Time PCR System (Applied Biosystems, USA). Primers GH1BF/GH1BR were designed to amplify partial conserved fragments of the family 1 β-glucosidase genes from bacteria, and primers GH3EF/GH3ER were designed to amplify partial conserved fragments of the family 3 β-glucosidase genes from fungi as previously described (Li, 2013). A tenfold serial dilutions of the linearized clone KY242445 containing the family 1 partial β-glucosidase gene fragments with a linear range of 3.29×10^5^ to 3.29×10^10^ copies μL^–1^ and linearized clone KY242451 containing the family 3 partial β-glucosidase gene fragments with a linear range of 2.26×10^5^ to 2.26×10^10^ copies μL^–1^ were used as a standard with R^2^ of 0.985 (efficiency=87.74%) and 0.935 (efficiency=84.74%), respectively. The internal reference gene and its standard curve range of the family 1 β-glucosidase genes from bacteria (GH1) and family 3 β-glucosidase genes from fungi (GH3) quantitative analysis shown in Table S1.

Each reaction was performed in a 20 µL volume containing 10 µL SYBR Premix Ex Taq (Takara, Dalian, China), 0.8 µM of each primer and 2 µL of 10-fold dilution DNA or cDNA template (1–10 ng). The qPCR thermocycling steps were as follows: 95 °C for 30 s, 45 cycles of 5 s at 95 °C, 34 s at 60 °C for family 1 β-glucosidase genes from bacteria (GH1) and family 3 β-glucosidase genes from fungi (GH3). Melting curve analysis and agarose gel electrophoresis confirmed the specificity of the amplification.

Li, H., Xu, X., Chen, H., Zhang, Y., Xu, J., & Wang, J., et al. (2013). Molecular analyses of the functional microbial community in composting by pcr-dgge targeting the genes of the β-glucosidase. *Bioresource Technology,* *134C*, 51-58.

| Primers of β-glucosidase gene | Clone fragment GenBank No. | Standard linear range（log copies g-1） | R^2^ | Amplification efficiency |
| --- | --- | --- | --- | --- |
| GH1B-a1F / GH1B-a1R | KY242445 | 3.297×10^3^〜3.29×10^10^ | 0.990 | 82.36％ |
| GH1B-b1F / GH1B-b1R | KY242446 | 2.90×10^3^〜2.90×10^9^ | 0.996 | 109.56％ |
| GH1B-c1 F/ GH1B-c1 R | KY242447 | 3.33×10^2^〜3.33×10^9^ | 0.993 | 82.51% |
| GH1B-d1F/ GH1B-d1 R | KY242450 | 3.15×10^3^〜3.15×10^10^ | 0.979 | 100.69% |
| GH3E-d3F/ GH3E-d3R | KY471087 | 2.21×10^3^〜2.21×10^10^ | 0.984 | 104.46% |
| GH3E-e3/ GH3E-e3F | KY471088 | 2.72×10^3^〜2.72×10^10^ | 0.988 | 91.96% |

Table S1 The internal reference gene and its standard curve range of family 1 β-glucosidase genes from bacteria (GH1) and family 3 β-glucosidase genes from fungi (GH3) quantitative analysis
